# Finished Annotated Genome Sequence of *Burkholderia pseudomallei* Strain Bp1651, a Multidrug-Resistant Clinical Isolate

**DOI:** 10.1128/genomeA.01427-15

**Published:** 2015-12-03

**Authors:** Julia V. Bugrysheva, David Sue, Janetta Hakovirta, Vladimir N. Loparev, Kristen Knipe, Scott A. Sammons, Satishkumar Ranganathan-Ganakammal, Shankar Changayil, Ganesh Srinivasamoorthy, Michael R. Weil, Roman L. Tatusov, Jay E. Gee, Mindy G. Elrod, Alex R. Hoffmaster, Linda M. Weigel

**Affiliations:** a Office of Infectious Diseases, Centers for Disease Control and Prevention, Department of Health and Human Services, Atlanta, Georgia, USA; b Office of the Chief Information Officer, Centers for Disease Control and Prevention, Department of Health and Human Services, Atlanta, Georgia, USA; c SRA International, Inc, Atlanta, Georgia, USA

## Abstract

*Burkholderia pseudomallei* strain Bp1651, a human isolate, is resistant to all clinically relevant antibiotics. We report here on the finished genome sequence assembly and annotation of the two chromosomes of this strain. This genome sequence may assist in understanding the mechanisms of antimicrobial resistance for this pathogenic species.

## GENOME ANNOUNCEMENT

*Burkholderia pseudomallei* is a Gram-negative bacillus and the etiologic agent of the potentially fatal, infectious disease called melioidosis (1). This saprophytic organism is considered endemic in Southeast Asia and Northern Australia. The infection usually occurs following contact with contaminated soil or water (1). *B. pseudomallei* strain Bp1651 was isolated from sputum of a hospital patient in the United States. This infection is likely to have been acquired in Australia, where the patient traveled several years prior. Antimicrobial susceptibility testing revealed that the strain was resistant to amoxicillin-clavulanic acid, ceftazidime, doxycycline, imipenem, and sulfamethoxazole/trimethoprim.

The *B. pseudomallei* Bp1651 genome was sequenced with the PacBio RSII system (Pacific Biosciences, USA). Eight single-molecule real-time (SMRT) cells were sequenced, and the data were assembled with the RS_HGAP_Assembly.3 protocol implemented in SMRT Portal version 2.3.0. There were a total of 243,100 subreads with an average length of 3,279 bp after filtering. A 30× long-read cutoff (6,316 bp) was used for preassembly, and the longest 15× of the corrected reads were assembled using Celera Assembler. The assembly resulted in two contigs (average depth of coverage 95.2) that correspond to the two chromosomes of this bacterium. We were able to close the contig corresponding to chromosome 1 into a circular chromosome based on the PacBio sequence assembly. The contig for chromosome 2 was closed into a circular chromosome using a paired-end option without addition of “N”s (see https://www.ncbi.nlm.nih.gov/assembly/agp/AGP_Specification for definition) by the National Center for Biotechnology Information (NCBI) Genome Submission group during submission of the sequence.

Even though the error rate for a single read with PacBio could be 12% or more, combining the multiple reads of the same region by the hierarchical genome-assembly process (HGAP) increases accuracy to 99.999%, as reported previously (2). The accuracy of the current HGAP3 assembly was verified by comparing the assembled genome to the whole-genome map generated by whole-genome mapping technology (OpGen Inc., USA) with *Bam*HI digest. We designated the sequence of the Bp1651 genome as finished (noncontiguous finished quality) because the ends of chromosome 2 were closed artificially (3).

Chromosome 1 is 4,112,209-bp long with a G+C content of 67.8%, while chromosome 2 is 3,147,816-bp long with a G+C content of 68.5%. These features are consistent with previously published *B. pseudomallei* genomes (4, 5).

The genome of *B. pseudomallei* Bp1651 was annotated using Prokka software (6). Chromosome 1 was predicted to contain 3,521 protein-coding sequences, 9 rRNA genes, and 59 tRNA genes. Chromosome 2 was predicted to contain 2,411 protein-coding sequences, 3 rRNA genes, and 26 tRNA genes. A predicted function was assigned to 75% of protein-coding genes. These numbers are similar to previously published genome annotations of *B. pseudomallei* (4, 5). We are currently analyzing sequences that may be associated with the antibiotic resistance of this strain.

### Nucleotide sequence accession numbers.

The finished annotated sequences of the two chromosomes of *B. pseudomallei* Bp1651 have been deposited at NCBI GenBank under the accession numbers CP012041 and CP012042.
